# The Friendship of Dr. Raue and Dr. Hering

**Published:** 1896-11

**Authors:** C. B. Knerr


### THE FRIENDSHIP OF DR. RAUE AND DR. HERING.

(An address delivered at the Raue Memorial Meeting at Hahnemann College, Philadelphia, October 17th, 1896, by Calvin B. Knerr, M. D.)

"A new and superb friendship."—WHITMAN.

"Happy is the house that shelters a friend."—EMERSON.

Shortly after Dr. Raue's death I received a letter from a friend and colleague which ended with the words: "I am sorry about Dr. Raue. I feel as though I had lost another part of Dr. Hering."

These words found a ready response within myself, for I had been long and intimately associated with both. What I personally owe to the influence of these two men I will not undertake to express. To have enjoyed the daily companionship of men like Hering and Raue; to have been admitted to their confidence and inmost thoughts; to have been allowed to work for them and with them most of us would consider a rare privilege, a liberal education in itself. I have always looked upon it as the luckiest thing of my life, a boon bestowed by Providence upon an unworthy recipient.

What, in my humble way, I can contribute to the history of the friendship of these truly great men, beacon-lights in the history of our cause, I look upon as a labor of love, a duty as pleasant as the placing of flowers upon the graves of loved ones.

Dr. Raue was twenty-eight years old when he met Dr. Hering, who was born twenty years before him. This disparity in their ages formed no obstacle to their friendship. Raue came as a pupil, full of desire to be filled with knowledge at a fountain ever flowing. Nature and environment had prepared the way for this meeting; "immense must have been the preparations for men like these, faithful and friendly the arms that helped them."

The little land of Saxony, so important on the map of Germany, had cradled both, had educated them to a period when their independent spirits longed for more freedom and a wider sphere of usefulness. Monarchical institutions were distasteful to them. They were alike strong in their love of freedom and their hatred of privileged classes. They felt themselves "cribbed, cabined, and confined," and longed for expression.

Hering sailed for South America at the age of twenty-six, Raue for the United States when twenty-eight. They were destined to meet here, in the City of Brotherly Love, where Hering had established Homœopathy after his pioneer work at Allentown, in this State.

Homœopathy had a good foothold, but needed energetic spirits to keep it alive. Good instructors were needed. The news of the good reception accorded Homœopathy in America had reached Germany. Raue heard of it and wanted to try his luck.

A letter of introduction from Dr. Hering's brother Carl, in Saxony, who knew young Raue well, was full of promise. To quote Hering's own words, taken verbatim from my notes: "Raue was assistant teacher to one by the name of Dressel, who was at the head of a higher institution of learning. Raue so highly distinguished himself in this capacity that Dressel wished to appoint him his successor, but Raue said: 'I want to go to America, and I want to study medicine.' There was a lack of funds. In order to obtain these he was advised to prepare a work for students on the *Psychology of Beneke*. He did so successfully, and made a sensation. He arrived in America and came to me. At first I advised him to study for the ministry. He got furious. 'I want to be a doctor, nothing else, and I want to be your student!' I thought to myself, this fellow will be either very much, or else he will amount to nothing at all, and adopted him."

A close relationship thus began, in 1848, between Hering and Raue, which ripened into a friendship to endure thirty-two years, until the time of Hering's death.

What the foundations for this more than ordinary friendship were I will endeavor to make plain by drawing parallel lines in the characters and characteristics of the two men.

In personal appearance they resembled each other. They were not men of ordinary mould. They were grand, sturdy men, of medium height and rugged athletic build. They gave the impression of being very much taller than they were in reality by reason of their commanding presence, their towering foreheads, leonine heads, and fine, expressive eyes.

Raue's head was round, had very expressive prominent frontal bones, the orbital region very well formed (fit receptacle for the philosopher's brain), the eyes deep-set, gray, fiery, sunny, sparkling; the hair blonde, later in life tinged with gray, and worn long like that of his friend and as was the custom with their race in the olden time. No doubt some thought that Dr. Raue imitated Dr. Hering. In fact, on one occasion, some one impertinently said to Dr. Raue: "You are the little Hering. You imitate him!" Dr. Hering, who was present, said: "No, you are mistaken; Raue does not imitate me. We are both THICK HEADS, that is all!"

It is a fact, however, the older Dr. Raue became the more he grew to resemble his friend in appearance; and this is not at all strange when we reflect how likeness in thought and soul-life shapes to similarity the features of those who live together long in close companionship and follow similar pursuits.

The grand head of Raue, set upon broad shoulders, assumed more and more the resolute and commanding look which people admired in Hering.

A workingman, in whose family Dr. Hering attended years ago, when engraving the block for Dr. Raue's portrait, to appear in one of the daily papers over an obituary notice, was struck with the resemblance and made the remark to his wife, "I made a picture of Dr. Hering to-day."

Small wonder that Raue should take on more and more the likeness of his friend when we come to realize that his whole world was bound up in him.

Daily, faithfully, and unfailingly, through all weather, Dr.

Raue paid his morning call to Dr. Hering, and Dr. Hering looked forward to it as to the rising of the sun. He would as soon have expected to have the one to fail him as the other, and surely Raue would have thought the planetary system out of joint if something had turned up to interfere with his visit to Dr. Hering.

In their dress both men were simple and unostentatious. Looks had to give way to comfort, fashion to common sense.

Their garments were worn loosely upon the body, their neckwear was not in the fashion, nor were their hats, of soft felt with wide brims, and their shoes were broad-soled and a world too wide to be in style, but easy and comfortable to the feet. In externals, as well as internals, their natures would not bear restraint; as Carlyle puts it, their "contempt for earthly shadows was always extreme."

Men like these "were never measured, and never will be measured." They were not "contained between their boots and hats."

In speech they were quite similar. Their language simple, natural, bold, and strong, free from sentimental rhetoric. Ordinarily they were placid and self-contained, but as is the case with men of great force, profound convictions, and energetic wills, they had a certain fierceness about opinions, in their minds indisputable, which they guarded jealously. When points like these were attacked, they became as explosive as dynamite, and their language was not always picked. They loved the truth and called things by their own names. Little cared they for consequences.

Relating to their utterance and tone of voice will apply the lines of Shakespeare:

> "His voice was propertied
> As all the tuned spheres, and that to friends;
> But when he meant to quail and shake the orb,
> He was as rattling thunder."
>
> —*Antony and Cleopatra.*

Both Raue and Hering could put up with personal affront. The moment the cause was attacked they were up in arms; like their countrymen, the fighting spirit was strong in them. The expressions called in German "Burschikos," the vigorous, one might say good, slangy expressions of the student-days, clung to them all their lives, and very refreshing they were to hear.

The spirit of satire, coupled with delicious wit, though drastic at times and biting when turned against an enemy to the cause, belonged as much to Raue as to Hering, although it is fair to say of Raue he never permitted much of it to get into his writings. He was no friend to polemics in literature.

Many a hard knock was dealt in this way by Hering, but only when deserved and in defense of the cause. It has been said, "All faults may be forgiven of him who has perfect candor," and no one will accuse either of our friends of ever being lacking in that.

Both were patient in listening. If you had an anecdote or story to tell, an observation to relate, or an experience to contribute, you were their man; neither one of them was ever in a hurry to see you go.

The moral and emotional side of the two men was phenomenal; they possessed courage, firmness, resolution; the will to dare and do in the highest degree, but controlled and seasoned and kept in bounds by the supremest *loyalty*—loyalty to friends, loyalty to family, loyalty to the cause. The tough fibre of the human heart was in their friendships.

Neither one cared for temporal prosperity as much as for things immortal. Not money-getters. No money consideration was ever an inducement, cold calculation an impossibility.

On one occasion Dr. Raue was seen coming from Hering's study in a state of great perturbation of mind, one might say high dudgeon. The cause of his disturbance was Dr. Hering himself, who, in an unguarded moment, had offered Raue a share in some money the latter had earned from Dr. Hering's patients while he was sick and unable to attend to practice.

When Raue had gone, Hering said: "Er ist ein göttlicher Grobian," which must have meant something very tender and complimentary, for Hering's eyes were moist when he said it!

Their integrity was beyond all doubt or scruple. They might have been, and sometimes, were deceived; but they never deceived others, for they had an inborn hatred of all that is mean, and never could tolerate shams or smart ways.

Dunham's words, spoken of Hering, apply to Raue as well: "The study of their lives was not fabrics, nor wares, nor stocks, but the noblest of God's creation, that which He made in His own image—the body and mind of man."

The words spoken of Hering by Henry N. Guernsey, are as true of Raue: "He never plotted evil, and never sought revenge, but was innocent-minded as a child." It must have been because in themselves dwelt this simplicity of heart, that their love and reverence for little children formed so marked a feature in their lives, stronger perhaps than any. If a child failed to smile for Dr. Hering, he pronounced it sick and in need of treatment! Dr. Raue was never happier than when he had little children, of his own or others, about him; his genial manner was then most genial, and when they were sick his kind heart overflowed with love and sympathy for them. In the sick-room he was then a welcome sight. The door would open. The house was brighter for his coming. He shed light like the sun. His hearty handshake, his merry laugh, his cheerful, healthy manner effused an atmosphere at once strengthening and saving. Hering had the same gift. Both men were strong believers in the saving power of optimism.

They took but little time for recreation and amusement, these two workers, not enough, and none for physical exercise as such. As some one expressed it, "their hygiene was very good, but it was for other people." Their industry, never flagging, was equalled only by their enthusiasm and power to endure.

That they both loved music goes without saying. They were Germans whose ears had become attuned from childhood to the best in music. To them were familiar and dear the chorales of Martin Luther, that sing of faith and hope, security and deliverance, eternal love and peace, and mighty praise such as armies offer up when the victory is won.

They loved well the music of Beethoven, never tired of hearing the septette, the sonatas, or the grander symphonies. Dr. Raue possessed a good tenor voice, with which he joined in singing the quartettes of Mendelssohn and other German four-part songs that were sung on birthdays and other festal occasions at either house.

They valued the excellency and nobleness of religion, these brave hearts, although they were not church-goers or worshipers in the accepted sense. They worked in the vineyard of the Lord with tenfold more earnestness than many who are. The mightiest are those in whom faith is mightiest. It was their custom to rail against dogma and empty form. If any one should happen to call Raue "a good Christian" in a sense a little distasteful to him, flaring up, he would say, "I am no Christian, and will not be called so in my own house!" On the other hand, if unobserved, he would be apt to go to the bedside of his little ones, tuck them in for the night, and tell them "to say their prayers to the dear God."

As to Homœopathy. As to the results of the concerted labor, the working methods, the teachings of these two friends, the sum total and outcome is incalculable. If Hering was a father to Homœopathy in this country, Raue was an elder brother to it. If Hering was an able general, Raue was a noble captain in the ranks. For thirty-two years the two worked together side by side, having the one thought uppermost in their minds, *to represent the master, and to represent him correctly*. They were Hahnemannians—they believed in the principles, and lived up to them.

Their working maxims were something like this: "There is an individuality in everything the Lord has made. You cannot substitute one medicine for another. To mix medicines is a crime. Alternating is the half-way house to mixing. To make a poor prescription, when much hurried, is excusable; the questions which always must be kept freshly in mind are: What is your aim? What are you striving for?" They were wont to say, "If a homœopathic physician once adopts the 'too-much-trouble creed' he is lost."

Their rules of practice—*golden* rules, they called them—

were: "Learn to observe. Learn to prove. Learn to examine the sick. Learn to select a remedy. Learn how to repeat and how to change remedies. Learn how to wait. Learn how to profit by experience."

They consulted their materia medicas diligently, they ransacked their repertories, unhandy and incomplete as they were. They added daily confirmations and new experiences to them. They possessed the ability to detect the individual characteristics of a remedy, and had an eye as well for the finer points of difference. They were artists in making prescriptions, Raue perhaps the greater therapeutist of the two.

Hering was a great promulgator of ideas, his fertile imagination constantly leading him on to new discoveries. When work was pressing Raue sometimes had all he could do to keep Hering from flying the track. He would say, "Hering is chasing a bee; I must bring him back!" If one was versatile the other was concentrated. They fitted together like two cogwheels, and kept the machinery in motion.

As teachers they were admirably fitted. Both had made teaching their occupation when young. Both loved to talk to students. They were ever ready to help young men. It made little difference to them who it was that came. If he had capacity to absorb he went away rich; but they never troubled themselves with the poor fact that the receiver was not capacious. Emerson says: "It never troubles the sun that some of his rays fall wide and vain into ungrateful space, and only a small part on the reflecting planet; let your greatness educate the crude and cold companion. If he is unequal he will presently pass away, but thou art enlarged by thine own shining."

Raue himself had been absorbing wisdom for nearly twenty years when, in 1867, his first homœopathic work, *Special Pathology and Therapeutic Hints*, appeared. It was dedicated to Hering in the following words:

"HONORED FRIEND:—As a token of most grateful acknowledgment of your uniform friendship so long enjoyed by me, and of my appreciation of your high attainments in science and vast experience in practice, I would dedicate to you this fruit of my humble labor.

"Your ever grateful

"PHILADELPHIA, December 3d, 1867." RAUE.

This book, marvelous in its completeness and practical scope, into the last edition of which (1896) the author put what was new in the progress of medical science, as well as the remainder of vitality left him, the writing of the preface being his last stroke of work upon earth, is a monument to his industry—surely a book no homœopath can afford to be without. It represents the making practical of what was theoretic, the showing of *how* the thing should be done. Hering inscribed his *Condensed Materia Medica*, likewise a text-book for students, to his friend Raue.

Raue's help in editing Hering's masterwork, *The Guiding Symptoms*, before and after Hering's death, was considerable. He not only contributed valuable material and advice, but he performed the arduous task of arranging and classifying the mental symptoms according to the system of psychology in which he was so well at home.

Let me say here that in respect to his later work, *Psychology Applied to the Solution of Occult Phenomena*, issued in 1889, Raue stands before the learned world an acknowledged master of his subject. To a homœopath it means that medicine has a spiritual side; that we cannot be successful physicians to the body without at the same time being physicians to the soul. It means that we shall make use of our ability, as Dr. Heerman, of Paris, expresses it, "to modify psychical tendencies in infancy and improve the race." Or what was foreshadowed in one of the theses of Hering's inaugural address, in 1826: "Not to deliver men from particular diseases, but to deliver the whole human race from the cause of disease is the ultimate goal of medical science."

As it was Hering's aim to elevate Homœopathy to a position among the sciences, as it was Beneke's effort to put mental philosophy on a firm ground, so it was Raue's purpose to continue that effort to a point where the human soul, "that being of which most men have but a shadowy idea, because they have never been accustomed to self-observation," may be estimated and measured according to the same law that develops the body, the law of affinity—*like attracts like.*

Men like Hering and Raue realized that the mind of our medical world, gross of perception and materialistic as it now is, is to be remedied by a gradual transformation. That Homœopathy should not be kept materialistic to adapt itself to the masses, but the masses must be educated to adapt themselves to it. It was their nature to proceed carefully; reasoning along the lines of inductive philosophy, setting firm ground for their ideas, waiting patiently for acknowledgment; realizing that—

> "All truths wait in all things,
> They neither hasten their own delivery nor resist it,
> They do not need the obstetric forceps of the surgeon."
> —WHITMAN.

Nevertheless, men like these wear themselves out in the service of humanity.

One evening Raue was called to minister to his friend, who was experiencing then "that bitter hug of mortality" to which he was prepared to say, "it is idle to try to alarm me." Even the trusty *Lachesis*, which had saved a thousand lives, could not save this one. His friend was no more.

The time had arrived when "a friendly, beckoning hand withdrew him from things without, his senses closed to page and speech, unfolded to sources of joy and hope, and he departed at peace with himself, with God, and the mantled world." *

Raue came next morning with bowed head, looked about the circle in which lay the dead friend, turned and went without a word, a broken-hearted man. He was unable to appear at the funeral.

No one understood Hering like Raue; and, I may say, no one Raue, like Hering.

Long after Hering had gone it was Raue's great delight to sit of an evening with a friend to whom the subject nearest his heart was congenial and talk about Dr. Hering and old times. Then he would become gloriously reminiscent, laugh, and be at his best; epithets rained, no end of adjectives.

---

*A correct estimate of Hahnemann.—C. HERING, 1847.

Hering was to Raue "philosopher, guide, and friend." Raue to Hering what he named him, "faithful Eckhardt;" more than this, a complement to the incomplete circle, a man with whom to live on brotherly terms.

As said Dunham, Raue could truthfully say, "In Constantine Hering I gained the most helpful, generous, and genial friend I have ever made." Dr. Hering could say, "In Raue I have never been disappointed."

Sixteen years after Dr. Hering had passed away death came as a loving friend to Dr. Raue. He was content to go, for his weak body had ceased to be an instrument to his capable will.

Some time back, when still in the possession of his faculties, upon one occasion when found upon his couch fatigued and in a fit of depression, such as is common to humanity, he is known to have said to his friend Hermann Faber, the artist, who, I think, stood next, after Hering, in his affections among his friends, "Dying is unpleasant, a miserable arrangement. If we but knew what is to come next!"

Referring to the *Psychology*, Faber answered him, "Open your book, Dr. Raue, and read what you have written!"

"Oh, that is all very well, as far as it goes, but we *know* nothing. Anyhow, you are a humbug!" With that he arose from his couch and passed into a pleasant humor.

At the last, when his spirit was clouded and he recognized no one about him, not even his dear "Mudding,"* he was heard to remark feebly, in German, "Es scheint mir es sind gerade 100 Jahre seit der gute Dr. Hering —" "It seems to me it is quite a hundred years ago since the good Dr. Hering — " The sentence remained unfinished. Apparently in his confused mind the centenary of Homœopathy, celebrated this year, commingled with memories of his beloved friend.

These were Raue's last words. A few days later his earthly body was consigned to flames, to be resolved into its elements and primitive forces.

---

* Pet name for his wife, taken from *Fritz Reuter's Stories.*

The flames his spirit have kindled will continue to burn brightly for the illumination of men's minds long after lesser lights have gone out.

These are the words of Emerson :—

"Let the soul be assured that somewhere in the universe it should rejoin its friend, and it would be content and cheerful alone for a thousand years."

They were brave, large, rough-hewn, of strong wide sympathies, these friends; believed in brotherhood, freedom, love, and hope. Are such as these destined to end in smoke and ashes?

What next? Will there not be another sunrise, more glorious than any?

These are the words of the Good Gray Poet :—

"This day before dawn I ascended a hill and looked at the crowded heaven,
And I said to my spirit, When we become the enfolder of those orbs, and the pleasure and knowledge of everything in them, shall we be filled and satisfied then?
And my spirit said, No, we but level that lift to pass and continue beyond."
—WHITMAN.

The intuitive and prophetic in us tell us that these comrades will continue their journey together: "They shall always persevere in the road which leads upwards."—*Plato.*

These are the words of the English poet, Symonds :—

"Morn now began to whiten in the wake
Of Phosphor: far athwart dim olive bowers
Freshened the breeze of dawning; so they rose.
As one with toil forespent, with waning powers,
Forth from the stifling city tumult goes,
In summer to fresh fields and hills serene,
For sure rejuvenescence and repose;
So toward the Alps and upland breezes keen,
The snows untroubled and the silver rills,
That death doth hide from life in his demesne,
Those comrades o'er the dew regenerate hills
Went smiling. Arm in stalwart arm enlaced,
Alike resplendent, and with wedded wills,
They seemed twin gods, fraternal stars embraced."

